# Estimating Common Growth Patterns in Juvenile Chinook Salmon (*Oncorhynchus tshawytscha*) from Diverse Genetic Stocks and a Large Spatial Extent

**DOI:** 10.1371/journal.pone.0162121

**Published:** 2016-10-03

**Authors:** Pascale A. L. Goertler, Mark D. Scheuerell, Charles A. Simenstad, Daniel L. Bottom

**Affiliations:** 1 School of Aquatic and Fishery Sciences, University of Washington, Seattle, Washington, United States of America; 2 Fish Ecology Division, Northwest Fisheries Science Center, National Marine Fisheries Service, National Oceanic and Atmospheric Administration, Seattle, Washington, United States of America; Maurice Lamontagne Institute, CANADA

## Abstract

Life history variation in Pacific salmon (*Oncorhynchus* spp.) supports species resilience to natural disturbances and fishery exploitation. Within salmon species, life-history variation often manifests during freshwater and estuarine rearing, as variation in growth. To date, however, characterizing variability in growth patterns within and among individuals has been difficult via conventional sampling methods because of the inability to obtain repeated size measurements. In this study we related otolith microstructures to growth rates of individual juvenile Chinook salmon (*O*. *tshawytscha*) from the Columbia River estuary over a two-year period (2010–2012). We used dynamic factor analysis to determine whether there were common patterns in growth rates within juveniles based on their natal region, capture location habitat type, and whether they were wild or of hatchery origin. We identified up to five large-scale trends in juvenile growth rates depending on month and year of capture. We also found that hatchery fish had a narrower range of trend loadings for some capture groups, suggesting that hatchery fish do not express the same breadth of growth variability as wild fish. However, we were unable to resolve a relationship between specific growth patterns and habitat transitions. Our study exemplifies how a relatively new statistical analysis can be applied to dating or aging techniques to summarize individual variation, and characterize aspects of life history diversity.

## Introduction

In Pacific salmon (*Oncorhynchus* spp.), diverse genetic stocks, morphologies and life histories spread mortality risk in time and space and can enable populations to respond independently to changes at a variety of spatial scales. This so-called “portfolio effect” may convey resilience in variable environments and stabilize abundance to help sustain healthy fisheries [1, 2]. In a conservation context, genetic and phenotypic diversity (e.g. life history diversity) is considered one of four important aspects for maintaining viable populations [3, 4]. Pacific salmon occupy a variety of freshwater, estuarine, and ocean habitats over the course of their lifetime, which supports variation in life-history traits. Adult salmon generally return to their natal streams and rivers to spawn, which produces genetically distinct populations and within species diversity due to their reproductive isolation. However, spatial variation in the freshwater and estuarine environment also decreases variation in juvenile survival over time, contributing to the “portfolio effect” [5, 6].

Juvenile life history types include different temporal and spatial patterns of habitat use, and are generally defined by their size relative to the age of migration or location of their rearing habitat in their natal watershed. A central dogma in salmon ecology and management often constrains juvenile life history diversity to ocean type and stream type juveniles, denoting whether individuals out-migrate to the ocean before or after their first year of life [7, 8]. However, this dichotomy involving one habitat transition and one migration age is an over simplification of juvenile life history rearing and out-migration behavior, that has been described in few systems [9, 10, 11]. By oversimplifying our descriptions of juvenile life history we may be ignoring other critical indicators of phenotypic diversity that are important to population resilience and long-term salmon conservation. Additionally, a narrow focus on timing and size at migration offers no direct measure of salmon performance. In this study we assess variations in growth to describe life history diversity and compare juvenile Chinook salmon (*Oncorhynchus tshawytscha*) performance by stock, migration route and timing.

Wild juvenile salmon life history diversity, especially estuarine entrance and residency can be difficult to document, and juvenile salmon life history diversity is not often integrated into resilience studies [5]. This may be due to the difficulty in tracking the migration of wild juvenile salmon and detecting their entrance into estuaries. Most tags used to track juvenile salmon movements and habitat use are too large to be used with the smallest size classes of juveniles (e.g., fry < 60mm FL), limiting the breadth of life history types that can be studied. The otolith chemical markers (e.g., Sr and Ca) most widely used to indicate fish entry into salt water are not limited by fish size, but provide no information about salmon rearing histories in the freshwater tidal reaches of estuaries [12]. This problem is particularly relevant for the Columbia River estuary, which is over 75% freshwater tidal [13]. There are currently insufficient chemical markers to distinguish the many tributary and freshwater estuary habitats from one another [14].

To address these knowledge gaps associated with freshwater tidal entry and the difficulty studying juvenile salmon life history diversity, we used a relatively new statistical technique to describe juvenile salmon diversity and simplify the sometimes overwhelming variation among individuals. Our objective was to identify the scope and scale of life history diversity among juvenile salmon by combining a variety of delineation methods, such as genetics, growth and origin. Specifically, we used Dynamic Factor Analysis (DFA) to describe common trends among time series of juvenile Chinook salmon growth rates [15]. Here, we take advantage of a unique genetic dataset [16] and collaborative effort on juvenile Chinook salmon occurrence in the tidal freshwater Colombia River estuary [17]. DFA has been used previously to identify patterns in abundance and productivity of adult salmon [18, 19], but we are unaware of any applications to juvenile salmon or individual dating or aging techniques, such as daily growth estimates derived from otolith microstructure.

We specifically focus on growth, which is a common metric for juvenile fish habitat quality [20–23], and varies between different types of nursery habitats for juvenile fish [24–27]. Furthermore, distinct changes in growth have been used to describe habitat changes in juvenile Chinook salmon [25, 28]. To address our objective, we used DFA to estimate common trends in those daily growth time series and the effects of any explanatory variables. In addition to the growth trends, we used genetic assignment to stock of origin as a proxy for the birthplace of each fish and capture location and date to reconstruct an individual’s freshwater journey through the Columbia River basin to the freshwater tidal reaches of the estuary.

## Methods

### Ethics Statement

This study falls under the auspices of National Oceanic and Atmospheric Administration (NOAA) and the University of Washington (UW). The research was conducted under NOAA’s Endangered Species Act (ESA) section 10 permits (Oregon Permit Numbers: 16148, 17021, 17890, Washington Permit Numbers: 10–433, 11–393, 13–024, and Federal Permit Numbers: 01-11-NWFSC81, 01-12-NWFSC81, 08-13-NWFSC81) and approved by UW’s Institutional Animal Care and Use Committee (Protocol Number: 2555–05). Lethal sampling was performed with the sedative MS-222, and all efforts were made to minimize suffering.

### Study System

The Columbia River basin extends into seven US states and one Canadian province; by volume it is the fourth largest river in the United States. The Columbia River has undergone significant modifications in its recent history. Cumulative development throughout the watershed has simplified Chinook salmon rearing habitat. These reductions may be an important factor in the apparent reduction in juvenile life history variation in Columbia River Chinook salmon [29]. Twenty-three mainstem and hundreds of tributary dams regulate the flow of the Columbia River. Dams without fish passage facilities have reduced access to spawning and rearing habitats, effectively eliminating 55% of the basin area historically available to salmon [30]. The installation of hydropower and irrigation diversion dams has had a significant impact on the timing and magnitude of the river discharge. For example, river regulation has reduced spring freshets and freshwater inputs to the estuary and effected estuarine circulation patterns [31]. Anthropogenic development of the Columbia River basin and estuary has extensively altered juvenile fish habitat. Diking, filling and other development in the estuary has noticeably reduced available rearing habitat [32] and altered the food base of much of the estuarine community [33]. Upland logging and agriculture, shoreline armoring, over-water structures, removal of large wood, and channel deepening and widening has progressively channelized and detached the estuary from its floodplain [34]. Today, estuary-wide loss of tidal riparian vegetation is estimated at 68% of the herbaceous tidal wetlands and 75% of forested tidal wetlands [35].

### Juvenile Chinook Sample Collection

**Fig 1:**
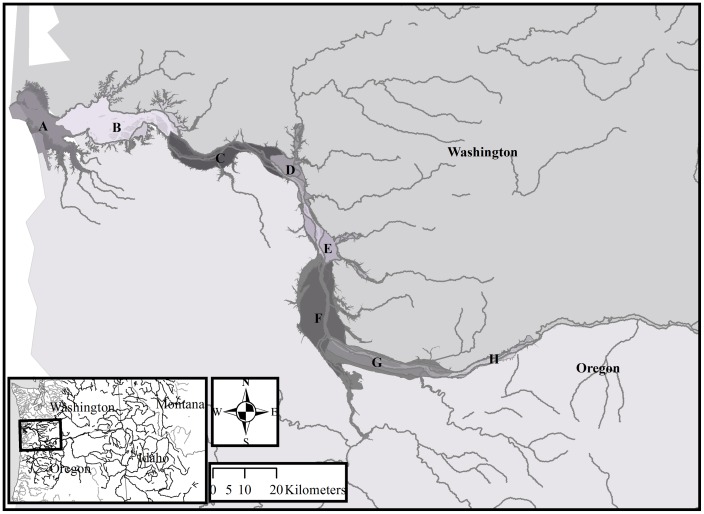
Columbia River basin (inset), and Level 3-Hydrogeomorphic Reaches (A-H) from the Columbia River Estuary Ecosystem Classification (adapted from Simenstad et al. [13]).

The samples used for this analysis were obtained from an estuary-wide survey to determine Chinook salmon genetic stock composition and distribution [16, 17]. Sites were chosen to characterize a range of environments in which both landscape scale influences (reaches) and as much stock and life history diversity as feasible could be captured (habitat types) specifically for a genetic survey, which is described in Teel et al. [16]. Sites were stratified among six freshwater tidal Level 3-Hydrogeomorphic Reaches (Fig 1: C-H) defined by the Columbia River Estuary Ecosystem Classification [13]. We sampled three habitat types within each reach: mainstem channel (M), backwater channel (B) and confluence (C). A total of eighteen sampling sites were sampled every-other month for two years (March 2010-March 2012) (Table 1). In all tables and figures, each site was coded by the sampling design, with the first letter representing the reach and the second letter representing the habitat type (e.g., the mainstem channel of reach C was CM). Beach seine sampling for juvenile salmon occurred down the full extent of each site with a 38-meter beach seine (1 cm mesh size), with a one-meter by one and a half-meter central bag (1/3 cm mesh size), which samples the top three meters of the water column. During flooding periods when beach seining was impractical, we used a 9-meter pole seine. Because small size classes of Chinook tend to favor shallow water habitats [36], this sampling design and method primarily targeted subyearling Chinook salmon. All fish caught were identified to species and counted. Up to 100 juvenile Chinook salmon were weighed and measured to the nearest millimeter (fork length). Fish were also scanned for coded wire tags (CWT), passive integrated transponder (PIT) tags and other markings (such as the removal of the adipose fin) to identify hatchery origin and other experimentally tagged groups. For each sampling event, tissue samples were obtained from the caudal fin for genetic analysis of up to 30 juvenile Chinook salmon, and preserved in nondenatured ethanol [36].

**Table 1:** A list of geographic coordinates for the study sites (adapted from Teel et al. [16]).

Site	Latitude (°)	Longitude (°)
CC	46.1902	−123.1247
CB	46.1684	−123.0808
CM	46.1707	−123.0730
DC	46.1034	−122.8954
DB	46.0901	−122.8989
DM	46.088	−122.9069
EC	45.8578	−122.7717
EB	45.7958	−122.7702
EM	45.7963	−122.7797
FC	45.6482	−122.7661
FB	45.7066	−122.7605
FM	45.7081	−122.7618
GC	45.5774	−122.4039
GB	45.5613	−122.4547
GM	45.5767	−122.4664
HC	45.6283	−121.9941
HB	45.6262	−122.0017
HM	45.6185	−122.0172

### Genetic assignment of fish to stock of origin

Probability estimates of individual assignment to stock of origin are reported in Teel et al. [16], and used in this study as a demographic descriptor (S1 Fig). Chinook salmon genotypes were determined using methods previously described in a Columbia River estuary study [37]. Genomic DNA was isolated from fin tissue samples using Wizard genomic DNA purification kits (Promega Corp.). The isolated genomic DNA was used in polymerase chain reactions (PCRs) to amplify 13 microsatellite loci, which have been standardized among several West Coast genetics laboratories [38]. GeneScan and Genotyper software programs (Applied Biosystems) were used to identify the size and number of alleles detected at each locus. The likelihood model described by Rannala and Mountain [39], and employed by the genetic stock identification program ONCOR [40], was used to estimate the stock origin of each individual. Population baselines correspond to a previously compiled multilaboratory standardized Chinook salmon genetic database [37, 41]. Fish were individually assigned to eleven regional stocks [16]. Nine regional stocks were identified as within basin (Table 2, Fig 1), and fish originating from the two out of basin groups (e.g., Rogue River and coastal populations) were not used in the otolith analysis. We chose to exclude out-of-basin stocks because they represented a small proportion of the juvenile Chinook salmon caught in this study, and it is unknown if they were the offspring of strays or juveniles migrating into the estuary from the marine environment.

**Table 2:** A list of the stock of origin abbreviations, their source tributaries within the Columbia River basin, and return timing of their stock of origin.

Abbreviation	Source Tributary(s)	Adult Return Timing
Desch_F	Deschutes River	fall
SCG_F	Spring Creek	fall
Snake_F	Snake River	fall
UCR_Su/F	Upper Columbia River	summer and fall
WC_F	West Cascade Range	fall and late fall
WC_Sp	West Cascade Range	spring
WR_Sp	Willamette River	spring

In addition, we used the GSI program ONCOR with the likelihood model of Rannala and Mountain [39] to compute the posterior probability of stock membership of each individual fish [42]. To ensure overall stock assignment accuracy in the data used for the DFA, we excluded juveniles with relative assignment probabilities < 0.80 (18% of the individual assignments determined from the total catch).

### Otolith Microstructure

Daily incremental growth was estimated for a subset of fish retained for otolith analysis (N = 665). These fish were opportunistically collected during several Columbia River estuary projects and therefore are not consistent over space or time (Table 3, S1 Fig). Daily incremental growth was estimated by measuring the microstructure features of each right sagittae otolith, viewed at adjacent transverse sections by two readers. The left sagittae otolith was used when the right was unavailable. Each otolith was mounted in Crystalbond resin, ground with fine grain sandpaper (1500 grit) and polished with MasterPrep^®^ Polishing Solution (Buehler, 0.05 micron) on both sides. Otoliths were photographed with a compound microscope (10x and 40x) and digital camera (Olympus B071). Otolith increments were counted and measured along transects 90° from the post-rostrum primordial in the dorsal direction [43]. For each transect, daily increment measurements originated at the exogenous feeding check [44] and extended to the otolith edge. Daily growth increments were measured and enumerated with ImageJ [45, 46] and a customized otolith and tree ring macro from the plugin ObjectJ (Developed by Vischer and Nastase, University of Amsterdam). Of the 665 otoliths dissected, approximately 5% (27) were lost due to damage during processing and 23 otoliths were determined to be vatritic, and therefore unusable. A relative index of reader confidence (1–5) was recorded for every otolith age estimate and only the highest rated samples (N = 524) were used for further analysis. To use otolith growth as a proxy for juvenile Chinook salmon growth, there must be a strong relationship between fish size and otolith size [47]. These data meet this necessary assumption with an R^2^ value of 0.95 when using otolith width to predict fork length. Growth time series were defined as the sequence of increment widths from emergence (exogenous feeding check) to capture in the estuary (otolith edge). Although there is the possibility of some error in the subjective nature of otolith microstructure aging methods, many of these errors are inversely related to one another [47]. Additionally, it has been suggested that aging fish with otolith microstructure by days lowers the risk of inaccuracies because of minor units of determination, and that the clarity of daily growth increments is most accurate in younger fish [47].

**Table 3:** Summary of the number of fish sampled by month, year, site, genetic stock of origin and hatchery (H) or wild (W) origin. Each site was coded by the sampling design, with the first letter representing the reach and the second letter representing the habitat type (e.g., the mainstem channel of reach C was CM).

Genetic Stock		Desch_F		SCG_F		Snake_F		UCR_Su/F		WC_F		WC_Sp		WR_Sp	
Origin		H	W	H	W	H	W	H	W	H	W	H	W	H	W
**May 2010**	CM										6				
	DB										15				
	DC										19		2		
	DM			1							18				1
	EM			4						1					
	FM			4						1	1				
	GM			5											
	HB		1	4	1		1		8		4				
	HC						1		12		2				
	HM			2	1				10	1	2				
**July 2010**	CB									2					
	DB								1	9	12				
	DC			2						20					
	DM				1					8	5		1		
	EB									1					
	FM							2							
	GB							1							
	GC									1					
	HB	1						5	11	1					
	HC		1					1	13		2				
	HM	1	1			1		5	6						
**September 2010**	DM									1	4				1
	EM								1		1				
	FM								3		1				
	GM								1						
**May 2011**	CM			2											
	DB			2							14				
	DC										14		1		
	DM			5					2		12				
	EB			1											
	FB			1											
	FC			2											
	GB		1		2		1		11		2				3
	GC								1		17				
	GM		1		1				16		4				
	HB			3	3		1		11						
	HM		1	1			1		12		3				
**July 2011**	CM									1					
	DB			2					1	9	6				
	DC									3	20		1		
	DM								2	2	18				
	EM							1							
	FB							1							
	FC					1		1							
	FM							1							
	GC									1					
	GM							1							
	HB							3	10						
	HM		2					6	6					1	
**September 2011**	HB				2			2	11				1		

### Growth Time Series

Due to high computation time we shortened each individual fish’s growth time series and the covariate time series by averaging over seven day periods. Summarizing variability over weeks is a common practice in otolith microstructure to incorporate growth variation and decrease the influence of edge effects [48, 49]. We also chose to only use growth estimates from one reader to decrease computation time; reducing estimation to a single **R** matrix per capture group. For 25% of the fish, each reader provided a second growth time series estimate, and had the exact same level of precision therefore we concluded they were interchangeable. To reduce computation time by removing series of NAs, the time series of daily individual juvenile Chinook salmon growth were grouped by capture month and year, totaling six capture groups: May 2010, July 2010, September 2010, May 2011, July 2011 and September 2011. Finally, these individual growth time series were standardized by z-scoring the absolute growth estimated by otolith microstructure.

### Dynamic Factor Analysis

We used dynamic factor analysis to model shared temporal trends in the daily growth increments among all fish, which allowed us to simplify the temporal variability in growth and observe the common trends. DFA is a multivariate time series method that seeks to explain the variance in *n* time series with a linear combination of *m* hidden random walks where *n* >> *m* [15]. The DFA model can be written as:

$$y_{t}=Zx_{t}+Dd_{i}+v_{t},$$

and

$$x_{t}=x_{t-1}+w_{t}$$

The *n* x1 vector of observations at time *t* (**y**_*t*_) are related to the *m* x 1 vector of latent trends at time *t* (**x**_*t*_) via the *n* x *m* matrix of factor loadings (**Z**). The *n* x *q* matrix **D** contains the effects of the *q* x 1 vector of covariates at time *t* (**d**_*t*_). The *n* x1 vector of observation errors at time *t* (**v**_*t*_) is distributed as a multivariate normal with mean-vector zero and variance-covariance matrix **R**. We assumed that otolith reader observation error would be equal for all fish, as the reader was blind to the identity of each otolith sample, and therefore all elements along the diagonal of **R** were unknown, but equal, and all off-diagonal elements were zero. The latent trends at time *t* (**x**_*t*_) were modeled as a random walk based on the process innovations at time *t* (**w**_*t*_), which are distributed as a multivariate normal with mean-vector zero and an identity variance-covariance (i.e., all diagonal elements were one with zeroes elsewhere).

We compared model fits based on one to five common trends for each of the capture groups. We chose five as the maximum number of trends because that was the number of juvenile Chinook salmon life history types described by Reimers [9]. We fit the models in R [50] using the package MARSS [51]. Support for each model was evaluated using Akaike information criterion, corrected for smalls sample sizes (AICc; [52]).

We hypothesized that growth increments for each individual fish would be affected by the environmental influences of their natal streams and the Columbia River estuary. Therefore, we included the **D** matrix in our DFA models to identify the influence of environmental covariates on growth variability over time. The **d** matrix included a water temperature time series for each of the major natal tributaries from which fish caught in this study had originated (Willamette River, Snake River, Deschutes River and Upper Colombia River), and the Columbia River estuary as well as discharge from the Columbia River estuary. We tested four different forms of **D** matrices. Each **D** matrix allowed for an individual’s genetic group to be affected by their natal stream temperature, and included the effects of Columbia River estuary temperature and discharge in four different ways. The first consisted of individual effects for each growth time series’ genetic reporting group with both Columbia River estuary temperature and discharge. The second constrained the effects for Columbia River temperature to be equal for all individuals. The third constrained the temperature effects to a single estimate for Columbia River discharge, and the fourth constrained both Columbia River estuary temperature and discharge to be the same for all individuals.

**Fig 2:**
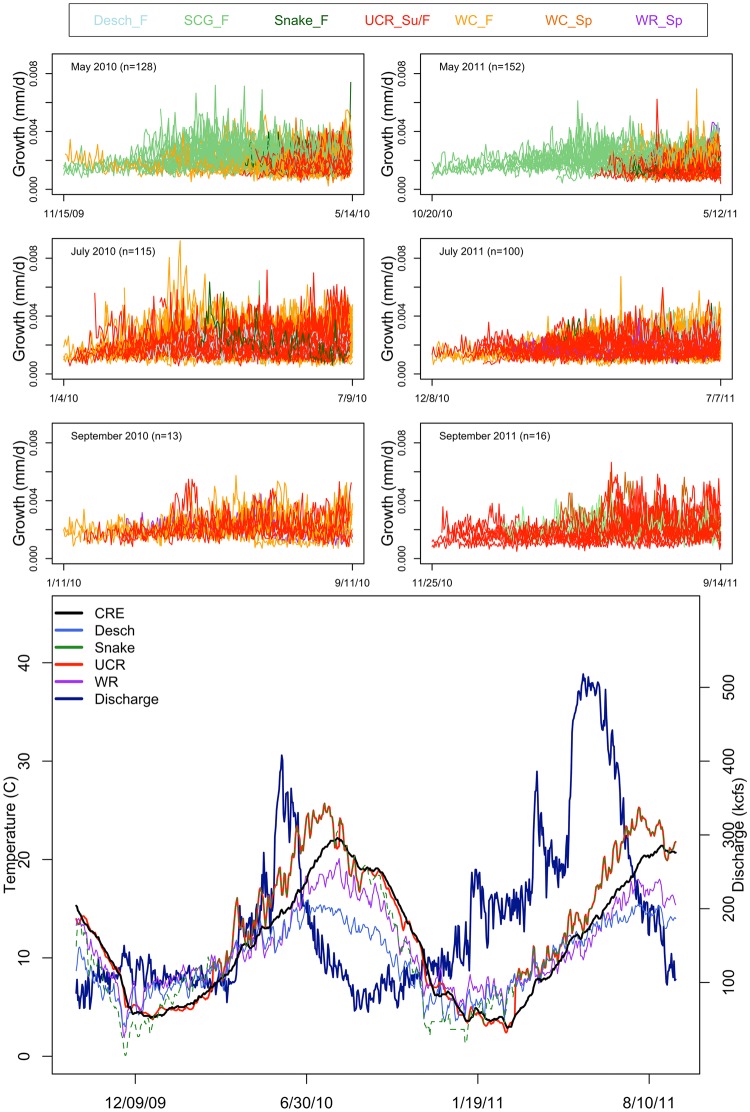
Growth time series for each individual juvenile Chinook salmon from estimated emergence date to capture in the Columbia River estuary (top six panels) and temperature and discharge covariate time series (bottom figure) used in the d matrix. Each time series was colored by genetic stock of origin (juvenile Chinook growth time series) and location (covariate time series). The covariate figure includes temperature from each major tributary and the Columbia River estuary temperature (CRE) and discharge (Discharge).

Measurements of daily average discharge and water temperature were obtained from the US Geological Service (Fig 2: CRE: Columbia River at Beaver Terminal, gauge number 14246900; WR: Willamette River at Portland, gauge number 14211720; Desch: Deschutes River near Culver, OR, gauge number 14076500; UCR: Columbia River Below Hwy 395 Bridge, gauge number 12514400; Snake: Snake River at Nyssa, OR, gauge number 13213100).

## Results

### Growth Time Series

Of the 524 otoliths used for growth time series, the largest sample sizes among capture groups were in May (2010: 128, 2011: 152) and July (2010: 115, 2011: 100), with September having much smaller sample sizes (2010: 13, 2011: 16). The length of individual time series (or fish estimated age) was generally proportional to fish length (R^2^ = 0.78). Fish length generally increased from May (average fork length was 53 mm), through July (70mm), to September (99 mm). In both years, fish size variability in May (coefficient of variation in fork length = 0.26) was greater than July (cv = 0.17) and September (cv = 0.13). Additionally, no one capture group represented all eighteen sampling sites (Table 3).

### Dynamic Factor Analysis

We used DFA to estimate common trends in individual growth time series and the effects of temperature and discharge in their natal basin. However associations to their genetic assignment to stock of origin, capture location and hatchery/wild origin were limited. We found varying support for consistent growth patterns among juvenile Chinook salmon. In general, we identified three major patterns: (1) increased growth over time, (2) decreased growth over time, and (3) one extreme period of increased or decreased growth between emergence and capture in the freshwater tidal estuary. The growth trajectories of juvenile Chinook salmon in capture groups May 2010, May 2011, July 2010, and July 2011 were best explained by one trend. However, juvenile Chinook salmon were best described by three and five trends, respectively in capture groups September 2010 and September 2011. In the DFA analysis, individual growth time series loaded both negatively and positively to one or more trends in each capture group, combining trends for individual fish and complicating interpretation.

Three and five DFA trends described in September as opposed to a single trend in May and July in both years may suggest increased diversity in juveniles which remained in freshwater through the fall or greater individual variation as fish age. Individuals captured in September were the oldest fish, and it is relatively intuitive that individual decisions could compound over time to create greater variance in growth as fish age. However, one artifact of being the oldest fish was that September capture groups had the longest growth time series and the smallest sample sizes (Table 3), and this could have affected our findings due to DFA’s method of modeling random walks. We found that September capture groups required much less computation time (as little as 3% of the computation required for May capture groups), which must be considered when interpreting the ecological implications of this result.

**Fig 3:**
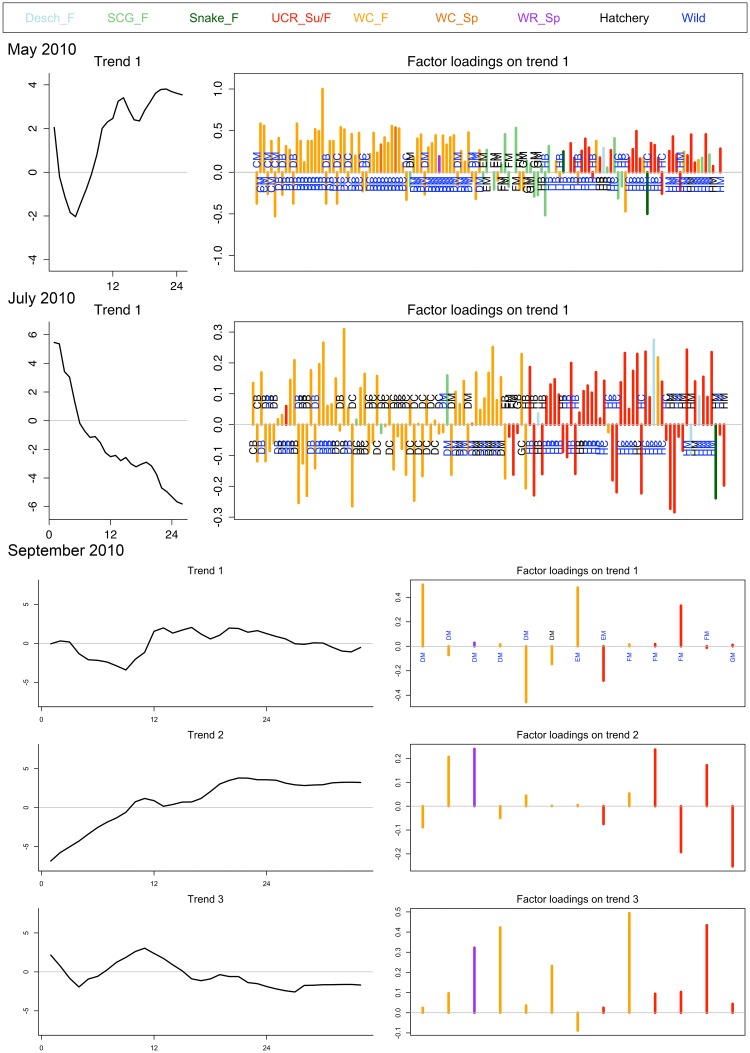
DFA trends (left) and trend loadings (right) for each monthly capture group in 2010. The trends are reported as z-scored growth rates (y-axis) over weeks (x-axis) starting at estimated emergence and ending at capture in the estuary. The trend loadings show each individual fish’s relationship to that trend (e.g. positive or negative) by capture location (site acronyms listed). The trend loadings also denote genetic stock of origin for each individual (bar color) and hatchery or wild origin (site acronym color).

The trend loadings of each individual juvenile Chinook salmon’s growth time series produced in the DFA analysis did not obviously align with genetic reporting group or capture location (see trend loadings in Fig 3). However, hatchery marked fish in May 2010 and to a lesser degree May 2011 capture groups had a narrower range of trend loading values (i.e. in May 2010 loadings ranged from 0.53 to -0.29 for hatchery fish and 1 to -0.53 for wild fish). Finally, the best covariate matrix was the **D** matrix with no covariates; it may be that growth at this individual scale cannot be adequately described by large scale temperature and discharge indices.

The DFA results from fish captured in 2010 varied by capture month. May 2010 exhibited an increasing pattern, with the majority of the fish loading positively (73%), suggesting higher growth later in life and closer to capture in the estuary (Fig 3). Hatchery fish loaded both positively and negatively to the May 2010 trend (11 negatively/12 positively), but operated within a smaller range of loading values. July 2010 displayed a decreasing pattern, with the majority of the fish loading positively (69%). Unlike any other capture period, the majority of the fish in July 2010 were hatchery fish (53%). The September 2010 capture group was best represented by three trends, two of which were pattern type three and one of which was an increasing pattern (Fig 3). The two type three trends had their extreme period of growth within the first twelve weeks post emergence and in opposing directions, and also had opposing loadings for the majority of those fish that loaded to both trends. Individuals loaded positively and negatively to all three trends; positively loadings were 43%, 55% and 88% for trend one (type three with decreased growth), two (increasing type) and three (type three with increased growth), respectively.

**Fig 4:**
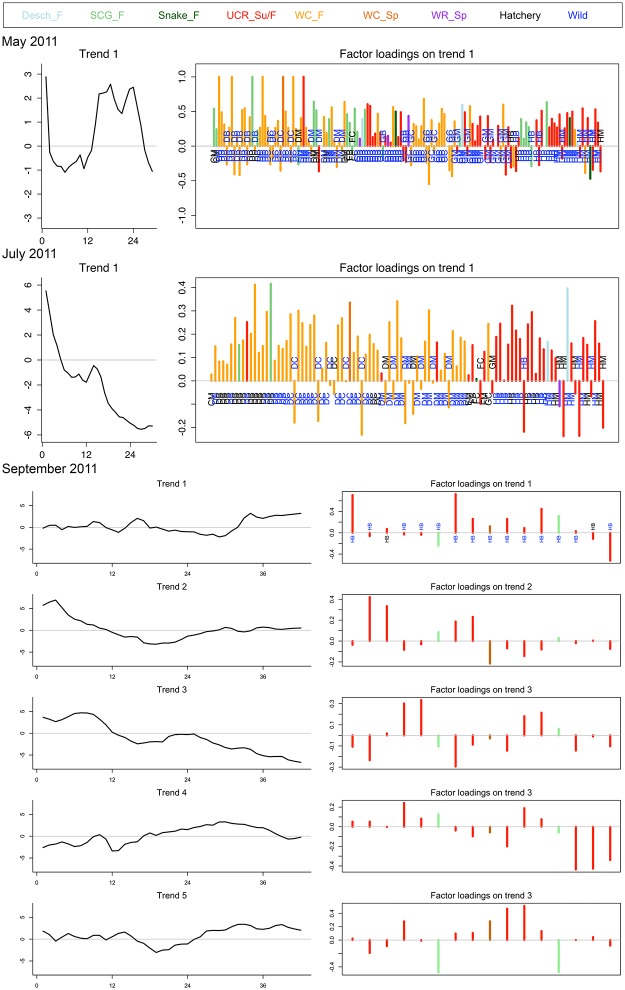
DFA trends (left) and trend loadings (right) for each monthly capture group in 2011. The trends are reported as z-scored growth rates (y-axis) over weeks (x-axis) starting at estimated emergence and ending at capture in the estuary. The trend loadings show each individual fish’s relationship to that trend (e.g. positive or negative) by capture location (site acronyms listed). The trend loadings also denote genetic stock of origin for each individual (bar color) and hatchery or wild origin (site acronym color).

Fish captured in 2011 likely encountered different environmental conditions in the estuary due to high discharge (Fig 2) in 2011, however DFA results are relatively similar by capture month to 2010. The trend representing fish in the May 2011 capture group indicates an extreme low growth period early in life followed by an extreme high growth period later in the time series (Fig 4). 73% of the fish loaded positively to this trend, with some particularly extreme positive loadings in fish captured in reach D. July 2011 exhibited a decreasing pattern, similar to July 2010. 82% of the fish in the July 2011 capture period loaded positively to this trend, with hatchery (34%) and wild fish loading similarly (Fig 4). Fish captured in September 2011 were best represented by five trends, which can be described by all three pattern types (Fig 4). All fish loaded to more than one trend with close to half the fish loading to all five trends (47%), which depicts unique combinations of the trends for each individual in September 2011.

## Discussion

Our study has documented a remarkable amount of individual growth variation among juvenile Chinook occupying the freshwater tidal reaches of the Columbia River estuary (Fig 2). Although we were unable to relate this diversity to our covariates, simply illuminating the diversity present has implications for salmon conservation. Regional stocks and capture location did not display divergent growth histories, but the DFA loadings did cluster by hatchery or wild origin for some capture groups, suggesting that hatchery fish may not experience the same breadth of growth variability as wild fish. We identified up to five large-scale trends in juvenile growth rates depending on month and year of capture. However, in some cases when interpreting the variability in trend loadings DFA produced as many growth patterns as individual fish. This study utilizes an innovative statistical technique to investigate aspects of juvenile life history diversity by integrating (1) growth trajectories, as a proxy for habitat transitions (2) time, and (3) demographics, such as origin. However, these results may be too complex for application in categorizing life history types, rather it better describes a continuum. This complexity in growth trends suggests salmon management should allocate resources which incorporate the use of the Columbia River freshwater landscape by a wide range of juvenile Chinook salmon at all times of year. Furthermore a focus on indicators for enhancing juvenile Chinook production basin-wide, rather than specific genetic groups is advisable, as there was no detectable relationship between estuarine growth [53] or life-time growth trends with stock of origin. Finally, other species and systems could benefit from innovative analyses which summarize individual variation to describe population-level diversity. DFA could be applied to otolith data from other teleost fish, dendrochronology and individual dating and aging techniques from many other disciplines. Analysis of biodiversity and resilience has been applied to a wide range of topics, such as the stability of human food systems, resource management for conservation and species-specific ecological studies [54, 55]. As a multivariate time series method, DFA is well adapted for resilience and diversity studies, which often document the asynchrony of time series.

Studies in smaller west coast basins have shown that estuaries may provide a critical contribution to salmon complexity as a mosaic of habitats connecting watersheds to the sea [56, 10]. Carl and Healey [56] described three life history types of Chinook salmon, which represent genetically distinct subpopulations adapted for juvenile rearing in the ocean, estuary and river. Each juvenile rearing habitat supported a separate metapopulation and aggregate biocomplexity. This has also been shown through habitat restoration; by the expression of a previously depressed juvenile life history type with the removal of dykes and the restoration of historic tidal marsh habitat for fry and fingerling Chinook [10]. In our study, all juvenile Chinook salmon examined with otolith microstructure and DFA are considered subyearlings or ocean-type by current fisheries standards for categorizing life history type [41]. However, we have shown diversity in growth history among these individuals. Similarly, some regional stock group’s capture months and sizes suggest a wide range of freshwater tidal occupancy [16] (Table 3, Fig 2, S1 Fig). For example, representatives of the Upper Willamette River stock were present in the freshwater tidal estuary most of the year (Table 3, Fig 2, S1 Fig). This group enters and exits the estuary at large sizes in March (more typical of the stream-type and what we may expect from a spring-run juvenile Chinook salmon), as well as very small (fry) sizes early in the year and as variable sizes of subyearlings throughout the late summer, fall and winter (Fig 2, S1 Fig). The West Cascade spring-run juveniles show a similar pattern, suggesting at least three estuary rearing or migrating time and size alternatives in lower river spring-run populations. Both lower river fall-run populations (West Cascade fall-run and Spring Creek group) and Upper Columbia River fall-run juvenile Chinook salmon are present in the freshwater tidal estuary at a variety of sizes year-round. These regional stock groups increase in size at capture from spring to late summer. Furthermore, summer months support the fastest growth of juvenile Chinook in the Columbia River estuary [53]. Therefore summer estuarine rearing may be important for all fall-run juveniles [57].

Despite this within-stock of origin variability in the catch data, we do not see a clear relationship with stock of origin and the growth trends produced from the DFA analysis (Figs 3 and 4). The DFA analysis best explained all capture groups by one to five trends. Similarly, the genetics, size and timing results combined descriptively suggest that a number of estuarine alternatives are present. However it remains unclear how growth and regional stock of origin relate to one another and influence life history diversity. It may be that many overlapping growth trajectories are present within any genetic stock, and that these growth trajectories represent relatively detailed variation from shifts in the duration of habitat transitions and resource availability for each individual. Evaluations of growth through bioenergentics include many variables, such as consumption rate, prey energy content, and habitat specific temperature [58, 59], which are not available at the landscape scale of this study. Individual age may also affect the variation estimated by growth trajectories and complicate the relationship with genetic group. Juvenile salmon captured in September were the oldest fish in our study and also exhibited more DFA trends than those captured in May or July. That is, growth may be constrained earlier in life and as individual decisions compound over time, growth variability may increase as an individual ages.

Even though the AICc values indicated a specific number of trend(s) were the most strongly supported for each capture group (Table 4), the variance in loadings represents individual variation around the large-scale trends in diversity. For example, some individuals within a capture group positively or negatively loaded to a single trend as well as (in the case of individuals captured in September) loaded to several trends. The complexity in which these individuals related to the produced trends creates individual versions of the combinations of trends, increasing the number of trends for interpretation. These results provide insight into how variable juvenile Chinook salmon freshwater rearing can be, and the many possible ways they could be categorized for conservation. Several other studies have also concluded that Chinook salmon may follow their life cycles through a variety of pathways, and there can be a tremendous amount of variation in juvenile use of and timing in estuaries [54, 60–65].

**Table 4:** Summary of the ΔAICc values for each number of trends and capture group without covariates. X denotes models that were not completed.

	# of trends				
	1	2	3	4	5
May 2010	0.00	207.04	549.39	X	X
July 2010	0.00	31.56	107.01	288.50	497.02
September 2010	62.12	26.68	0.00	8.79	13.94
May 2011	0.00	195.56	635.23	X	X
July 2011	0.00	35.87	95.77	204.74	426.65
September 2011	31.14	15.18	13.76	9.48	0.00

In addition, the growth trends estimated by DFA do not have a clear relationship with hydrogeomorphic reach or habitat type in which they were captured in the freshwater tidal estuary (Figs 3 and 4). This may in part be due to sampling design. The otolith samples were an opportunistic subsample of a larger study that did not collect an equal number of otoliths at every capture event in all 18 sites. Furthermore, the **D** matrix with no covariates was the most supported by AICc. Therefore, we were unable to relate individuals, genetic groups or trends to specific environmental variables in the estuary or natal tributaries. Unfortunately, without a distinction in growth between the estuary and natal tributary we cannot assign particular attributes to any evidence of estuarine rearing. Additionally, other demographic changes, such as a release from gape limitation or pulses of high caloric prey items (e.g., larval fish) could be affecting our growth estimates. Without the availability or integration of this metabolic information the scale of variation (e.g. daily growth) may be too in depth to make generalizations about estuarine or fluvial habitat use with DFA.

Regional stocks and capture habitat did not display consistent growth patterns, but the marked hatchery fish did have a smaller range of trend loadings from the DFA analysis in May capture periods. Additionally some of the hatchery marked individuals indicated a pattern we may predict from the known portion of their time rearing in freshwater: high initial growth (potentially when fed in the hatchery environment) followed by a steep but variable decline in growth and eventual leveling off (representing their transition into a wild more challenging environment) (Fig 3: July). If a proportion of an individual hatchery fish’s life is experienced in a controlled environment it is plausible that it would experience less variability, despite being caught in the wild. These results suggest that hatchery fish may not experience the same breadth of growth variability as wild fish. Sampling lower in the Columbia River estuary has shown that hatchery-produced fish have limited variation in timing and abundance [66].

Finally, the dynamic factor analysis reveals considerable diversity in juvenile Chinook salmon but may not be a practical approach for identifying juvenile life history diversity management targets, due to the complexity of the results and time intensive laboratory and statistical techniques. It is important to continue to investigate methods for identifying freshwater tidal estuarine residence [67]. Nearly $170 million is spent annually to artificially enhance the survival of juvenile Columbia River salmon populations in the freshwater phase of their life cycle (e.g., hatchery production and more natural habitat processes) [68]. To counteract harvest declines and ameliorate rearing habitat loss, hatcheries target fast growing and large-sized juveniles. Although growth has been shown to be an important indicator for juvenile salmon survival [69], there is no one optimum phenotype. Therefore, a better understanding of juvenile salmon life history diversity could further convey the importance of diversity and supply a target for hatchery management, permitting requirements, habitat restoration and hydropower practices, which could work towards allowing an expression of diversity instead of producing a single phenotype [70]. Habitat restoration efforts could also integrate habitat complexity as a means for promoting species resilience, if juvenile habitat use could be better estimated and applied. Restoration efforts in the Salmon River estuary have shown that wetland recovery can expand life history variation by allowing a greater expression of estuarine-resident behaviors [10]. However, few studies incorporate a landscape scale evaluation of habitat performance, and genetic and life history diversity that can be integrated into restoration design. Therefore, a targeted and research based approach to building a restoration framework focused on landscape scale juvenile Chinook diversity and habitat quality across a range of estuarine wetland habitats could advance salmon management. Many of the Columbia River Chinook salmon populations are listed as threatened or endangered on the Endangered Species Act, and managers and habitat restoration practitioners have a responsibility to integrate diversity and resilience into salmon management and conservation as we prepare for the consequences of climate change on our natural resources.

## Supporting Information

S1 Fig The distribution of size (mm), timing and genetic group of juvenile Chinook salmon over space and time in the Columbia River estuary (adapted from Teel et al. 2014). The inner grid represents space: the intersection of each line is one of the 18 locations from which we sampled. The outer grid represents time: each larger grid cell is a combination of month (January to November) and year (2010–2012).
(TIF)
