# Supplementary figures and images for: Estimating Common Growth Patterns in Juvenile Chinook Salmon (Oncorhynchus tshawytscha) from Diverse Genetic Stocks and a Large Spatial Extent
Source: PLoS One. 2016 Oct 3;11(10):e0162121. doi: 10.1371/journal.pone.0162121 (PMC5047595; doi:10.1371/journal.pone.0162121)

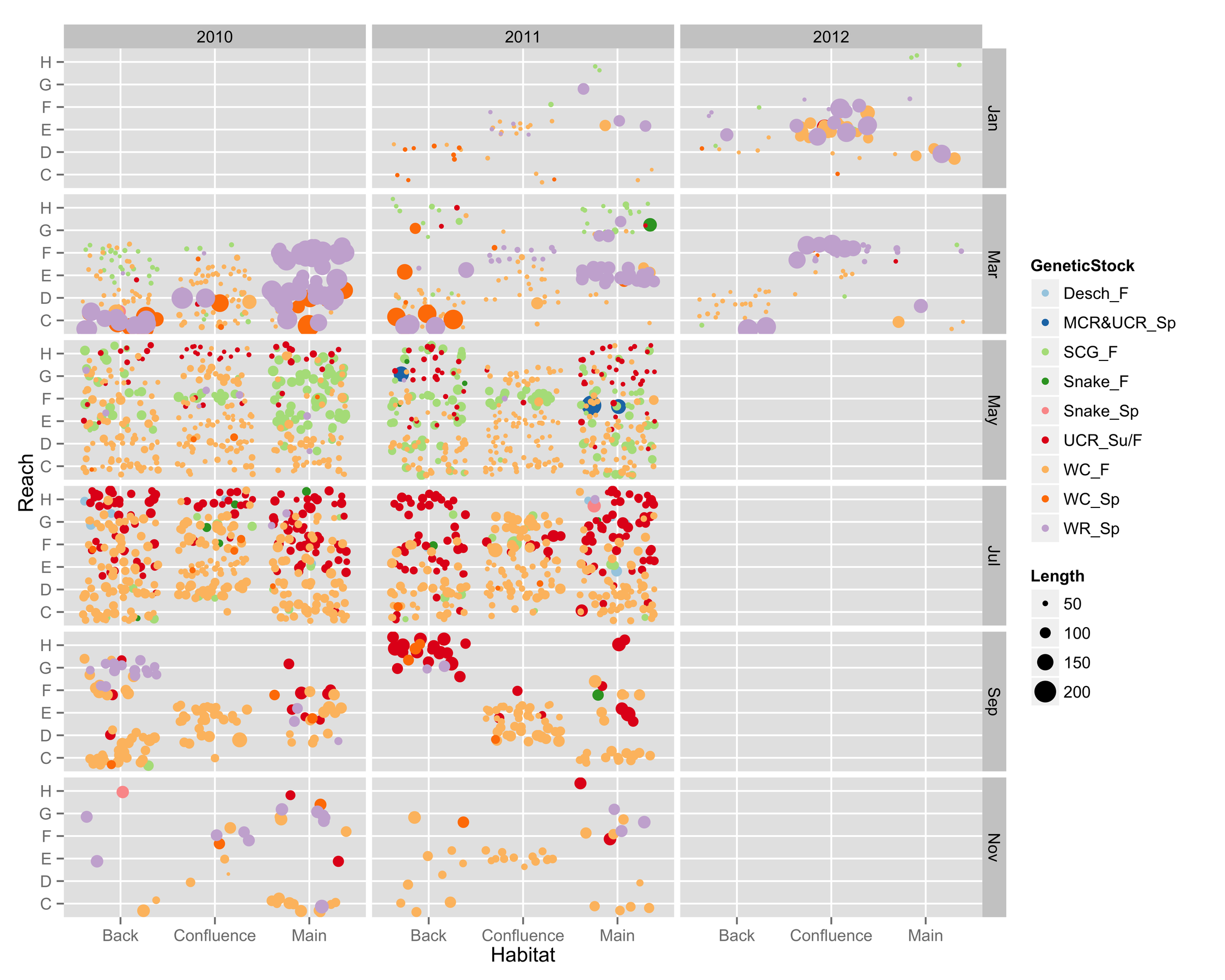

Supplement: S1 Fig — The inner grid represents space: the intersection of each line is one of the 18 locations from which we sampled. The outer grid represents time: each larger grid cell is a combination of month (January to November) and year (2010–2012). (TIF) [file pone.0162121.s001.tif]
